# Development and validation of a quadruplex real-time quantitative polymerase chain reaction assay for the simultaneous detection of Mycoplasma hyopneumoniae, Mycoplasma hyorhinis, Actinobacillus pleuropneumoniae, and Glaesserella parasuis

**DOI:** 10.14202/vetworld.2026.3944-3960

**Published:** 2026-09-11

**Authors:** Hongyu Wei, Yu Gan, Kaichuang Shi, Yuwen Shi, Feng Long, Shuping Feng, Yanwen Yin, Sujie Qu, Wenjun Lu, Yingyi Wei

**Affiliations:** 1 College of Animal Science and Technology, Guangxi University, Nanning 530005, China.; 2 Guangxi Center for Animal Disease Control and Prevention, Nanning 530001, China.

**Keywords:** Actinobacillus pleuropneumoniae, Glaesserella parasuis, multiplex real-time quantitative polymerase chain reaction, Mycoplasma hyopneumoniae, Mycoplasma hyorhinis, porcine respiratory disease complex, swine, TaqMan probe

## Abstract

**Background and Aim:**

*Mycoplasma hyopneumoniae*, *Mycoplasma hyorhinis*, *Actinobacillus pleuropneumoniae*, and *Glaesserella* *parasuis* are major bacterial pathogens associated with porcine respiratory disease complex (PRDC) and frequently occur as single or mixed infections, causing substantial economic losses to the swine industry. Because these pathogens produce similar clinical signs and pathological lesions, rapid and accurate differential diagnosis is essential for effective disease control. Conventional single-target assays are labor-intensive and less suitable for simultaneous pathogen detection. Therefore, this study aimed to develop and validate a quadruplex real-time quantitative polymerase chain reaction (qPCR) assay for the simultaneous detection of these four important porcine respiratory bacterial pathogens and evaluate its applicability using clinical samples.

**Materials and Methods:**

Four pairs of species-specific primers and TaqMan probes targeting the *p97* gene of *M. hyopneumoniae*, *p37* of *M. hyorhinis*, *apxIV* of *A. pleuropneumoniae*, and *ompA* of *G. parasuis* were designed. The reaction system and amplification conditions were optimized, followed by evaluation of analytical performance, including linearity, sensitivity, specificity, and repeatability. A total of 1,238 clinical samples (821 lung tissues and 417 nasal swabs) collected from pigs in Guangxi Province, China, were tested using the developed assay and reference methods. Diagnostic sensitivity, diagnostic specificity, agreement rates, and pathogen prevalence were determined.

**Results:**

The optimized quadruplex assay showed excellent linearity, with correlation coefficients (R²) ≥0.998 for all four targets. The limits of detection were 152.02, 158.53, 161.36, and 140.40 copies/reaction for *M. hyopneumoniae*, *M. hyorhinis*, *A. pleuropneumoniae*, and *G. parasuis*, respectively. No cross-reactivity with other common swine pathogens was observed, demonstrating high analytical specificity. The assay also showed excellent repeatability, with intra- and inter-assay coefficients of variation below 2.50%. Among the 1,238 clinical samples, the positivity rates were 31.91% for *M. hyopneumoniae*, 18.09% for *M. hyorhinis*, 4.20% for *A. pleuropneumoniae*, and 26.82% for *G. parasuis*. Mixed infections were detected in 19.71% of samples, with *M. hyorhinis* + *G. parasuis* representing the most frequent co-infection (11.79%). The assay demonstrated diagnostic sensitivities of 96.77%, 95.95%, 95.92%, and 95.87% and diagnostic specificities of 99.40%, 98.92%, 99.58%, and 99.22% for *M. hyopneumoniae*, *M. hyorhinis*, *A. pleuropneumoniae*, and *G. parasuis*, respectively, with agreement rates exceeding 98% for all pathogens.

**Conclusion:**

The developed quadruplex real-time qPCR assay provides a rapid, sensitive, specific, and reproducible method for the simultaneous detection of four major bacterial pathogens associated with PRDC. Its excellent diagnostic performance and ability to identify mixed infections make it a valuable tool for routine laboratory diagnosis, epidemiological surveillance, and disease control in pig populations, with potential application in large-scale surveillance programs.

## INTRODUCTION

Porcine respiratory disease complex (PRDC) is one of the most important and costly health problems affecting commercial swine production worldwide because it results from the synergistic interactions of multiple pathogens [1]. Previous studies have reported that the median economic impact of endemic respiratory pathogens ranges from €1.70 to €8.90 per nursery pig, €2.30 to €15.35 per fattening pig, and €100 to €323 per sow annually [2]. Furthermore, severe lung lesions associated with PRDC can reduce carcass weight by 2.29–2.77 kg, resulting in financial losses of up to €11.53 per 100 kg of meat [3]. Among the bacterial pathogens associated with PRDC, *Mycoplasma hyopneumoniae* (Mhp) is recognized as the primary pathogen, whereas *Actinobacillus pleuropneumoniae* (APP) and *Glaesserella* *parasuis* (Gps) are important secondary bacterial pathogens that directly contribute to disease progression [4, 5]. Although *Mycoplasma hyorhinis* (Mhr) is not considered a primary etiological agent of PRDC, it may indirectly contribute to disease development by impairing host immunity and exacerbating respiratory disease [6].

In China, the pooled prevalence of Mhp is 33.4% (95% confidence interval [CI]: 26.5%–40.7%), increasing to 52.9% in clinically affected farms [7]. The reported detection rate of Mhr is 31.77% [8], whereas the pooled prevalence of Gps is 27.8% (95% CI: 22.6%–33.0%) [9]. In contrast, the detection rate of APP is relatively low, ranging from 0.45% to 5.43% [10, 11], although mixed infections are frequently reported [10, 11]. Because these pathogens produce similar clinical manifestations, including coughing, fever, and arthritis, accurate differentiation based solely on clinical presentation is challenging [12, 13]. In addition, co-infections frequently result in overlapping clinical features, further complicating disease diagnosis and control. Conventional diagnostic methods, such as bacterial culture, polymerase chain reaction (PCR), and enzyme-linked immunosorbent assay (ELISA), are time-consuming, have limited sensitivity, or cannot distinguish antibodies induced by natural infection from those induced by vaccination [4, 14, 15].

Although several multiplex PCR or quantitative PCR (qPCR) assays capable of detecting two or three of these pathogens have been reported [16–18], no multiplex assay has been developed for the simultaneous detection of all four major bacterial pathogens associated with PRDC, namely Mhp, Mhr, APP, and Gps. The absence of such an assay limits the efficiency of differential diagnosis, particularly in cases of mixed infections, which are common in pigs with respiratory disease. Therefore, there remains a need for a rapid, sensitive, specific, and high-throughput diagnostic assay capable of simultaneously detecting these four clinically important pathogens in a single reaction.

Multiplex qPCR enables the simultaneous detection of multiple targets in a single reaction. Compared with conventional singleplex qPCR assays, this approach offers several advantages, including an approximately 75% reduction in reagent consumption, a turnaround time of approximately 2 h, and a substantially lower risk of missing co-infections, making it well suited for the simultaneous screening of pathogens with similar clinical presentations [15, 19].

Therefore, this study aimed to develop and validate a quadruplex qPCR assay for the simultaneous detection and differentiation of Mhp, Mhr, APP, and Gps. Four pairs of species-specific primers and four TaqMan probes were designed based on multiple sequence alignments of the *p97* gene of Mhp, the *p37* gene of Mhr, the *apxIV* gene of APP, and the *ompA* gene of Gps from isolates reported in different countries. The analytical sensitivity, specificity, repeatability, and clinical applicability of the developed assay were subsequently evaluated to determine its suitability as a rapid, sensitive, cost-effective, and high-throughput diagnostic tool for the differential diagnosis and epidemiological surveillance of PRDC-associated bacterial infections. To the best of our knowledge, this is the first quadruplex qPCR assay capable of simultaneously detecting these four PRDC-associated bacterial pathogens in a single reaction, thereby addressing an important diagnostic gap and facilitating the identification of mixed infections.

## MATERIALS AND METHODS

### Ethical approval

The study protocol was reviewed and approved by the Guangxi Center for Animal Disease Control and Prevention (CADC), China (Approval No. 2020-A-02), on November 15, 2020, before the commencement of the study and collection of clinical samples. The study involved the collection of lung tissue and nasal swab samples from pig farms and slaughterhouses in Guangxi Province, China. Written informed consent was obtained from the animal owners before sample collection. All sampling and experimental procedures were conducted in accordance with the applicable institutional guidelines for animal welfare and ethical use of animal-derived materials. Appropriate measures were taken during sample collection and handling to minimize unnecessary stress or discomfort to the animals.

### Study period and location

This study was conducted at the Guangxi Center for Animal Disease Control and Prevention (CADC), Nanning, China, between February 1, 2025, and April 30, 2026. All experimental procedures were performed in accordance with the Minimum Information for Publication of Quantitative Real-Time PCR Experiments (MIQE) guidelines.

### Viral and bacterial strains

The vaccine strains of pseudorabies virus (PRV; strain Bartha-K61) and swine influenza virus (SIV; strain TJ) were purchased from Wuhan Keqian Biology Co., Ltd., Wuhan, China. Porcine circovirus type 2 (PCV2; strain DBN-SX07) was obtained from Sichuan Hailinge Biopharmaceutical Co., Ltd., Chengdu, China. Porcine reproductive and respiratory syndrome virus (PRRSV; strain TJM-F92) was obtained from Jilin Teyan Biotechnology Co., Ltd., Changchun, China.

The vaccine strains of Mhp (strain J), *Escherichia coli* (trivalent vaccine containing strains SD04, HN03, and JS01), group C *Streptococcus* (strain BHZZ-L1), *Streptococcus suis* serotype 2 (strain BHZZ-L4), Gps (quadrivalent vaccine containing serotype 4 strain SD02, serotype 5 strain HN02, serotype 12 strain GZ01, and serotype 13 strain JX03), and *Salmonella choleraesuis* (attenuated strain C500, CVCC79500) were purchased from Shandong Huahong Bioengineering Co., Ltd., Jinan, China. The vaccine strains of classical swine fever virus (CSFV; strain C), *Erysipelothrix rhusiopathiae* (strain G4T10), *Pasteurella multocida* (strain EO630), and APP (trivalent vaccine containing serotype 1 strain JL9901, serotype 2 strain XT9904, and serotype 7 strain GZ9903) were obtained from China Animal Husbandry Industry Co., Ltd., Beijing, China. Positive samples of Mhr were provided by our laboratory. Detailed information on all vaccine strains and positive samples is presented in Supplementary Table S1.

### Clinical samples

Clinical samples were collected between March 1, 2025, and February 7, 2026. A total of 821 lung tissue samples were collected from 19 slaughterhouses located in 14 cities of Guangxi Province, China. In addition, 417 nasal swab samples were collected from 11 slaughterhouses and 36 commercial pig farms.

Lung tissue samples were collected from pigs exhibiting pulmonary consolidation and/or carnification, whereas nasal swab samples were obtained from pigs showing clinical signs of dyspnea and/or coughing. Pigs sampled at slaughterhouses were >8 months of age, whereas pigs sampled from farms included weaners, finishers, and sows of different ages. All pigs had been vaccinated with an inactivated Mhp vaccine but had not been vaccinated against Mhr, APP, or Gps.

All samples were transported to the laboratory under refrigerated conditions (≤4°C) within 6 h after collection and were stored at −80°C until further analysis.

### Primers and probes

Multiple sequence alignments of 31 *p97* gene sequences from Mhp, 35 *p37* gene sequences from Mhr, 46 *apxIV* gene sequences from APP, and 78 *ompA* gene sequences from Gps were retrieved from the National Center for Biotechnology Information (NCBI) GenBank database (https://www.ncbi.nlm.nih.gov/nucleotide/; accessed February 13, 2025). Detailed information on the reference strains is provided in Supplementary Tables S2–S5.

Based on conserved regions identified through sequence alignment, four pairs of species-specific primers and corresponding TaqMan probes were designed using Oligo 7.0 software (https://www.oligo.net/ downloads. html). The specificity of the designed primers and probes was verified using the Basic Local Alignment Search Tool (BLAST) available through the NCBI GenBank database (https://blast.ncbi.nlm.nih.gov/Blast.cgi; accessed February 13, 2025). The BLAST results for all primers and probes are summarized in Supplementary Table S6.

The primer and probe sequences are presented in Table 1, and their target locations are illustrated in Figure 1. Primers and probes were synthesized by IGE Biotechnology Co., Ltd., Guangzhou, China. Primers were purified by polyacrylamide gel electrophoresis, whereas probes were purified by high-performance liquid chromato-graphy.

### DNA extraction

Approximately 0.3 g of lung tissue was transferred to a 2.0 mL centrifuge tube containing phosphate-buffered saline (PBS; pH 7.2; tissue:PBS ratio = 1:4, w/v) and sterile steel beads. After three freeze-thaw cycles, the samples were homogenized using a tissue homogenizer (Retsch, Haan, Germany) and centrifuged at 13,500 × *g* for 1 min at 4°C using a refrigerated benchtop centrifuge. The supernatant was collected for nucleic acid extraction.

**Table 1:** Primers and probes used for the quadruplex quantitative polymerase chain reaction assay.

**Primer/probe**	**Sequence (5′→3′)**	**Product size (bp)**
Mhp-p97-F	ACAGACTAAATAATGCTCCTGA	158
Mhp-p97-R	CGGGCACTTTGACTAAGAT	
Mhp-p97-P	FAM-AGTATCCAGAACCAAATTCCTTCGC-BHQ1	
Mhr-p37-F	GCAGATACATTGGGAACTTTAGA	165
Mhr-p37-R	CACTCCAACATCATACGGAATT	
Mhr-p37-P	ROX-TGAACACATAACAAATCAGCAACAAAACCT-BHQ2	
APP-apxIV-F	CGTTGCCGCCCATTTATC	216
APP-apxIV-R	AACCCGTTTTATAGCCGAT	
APP-apxIV-P	VIC-AAGCAGCCAACTCCTCAGAAAGTG-BHQ2	
Gps-ompA-F	ATGCTGCCAACTTAACTCT	162
Gps-ompA-R	AATGCTTTACGACCTTTAACTGC	
Gps-ompA-P	CY5-AAAACGTGCAGAAACAGTAGCAAAC-BHQ2	

**Figure 1:**
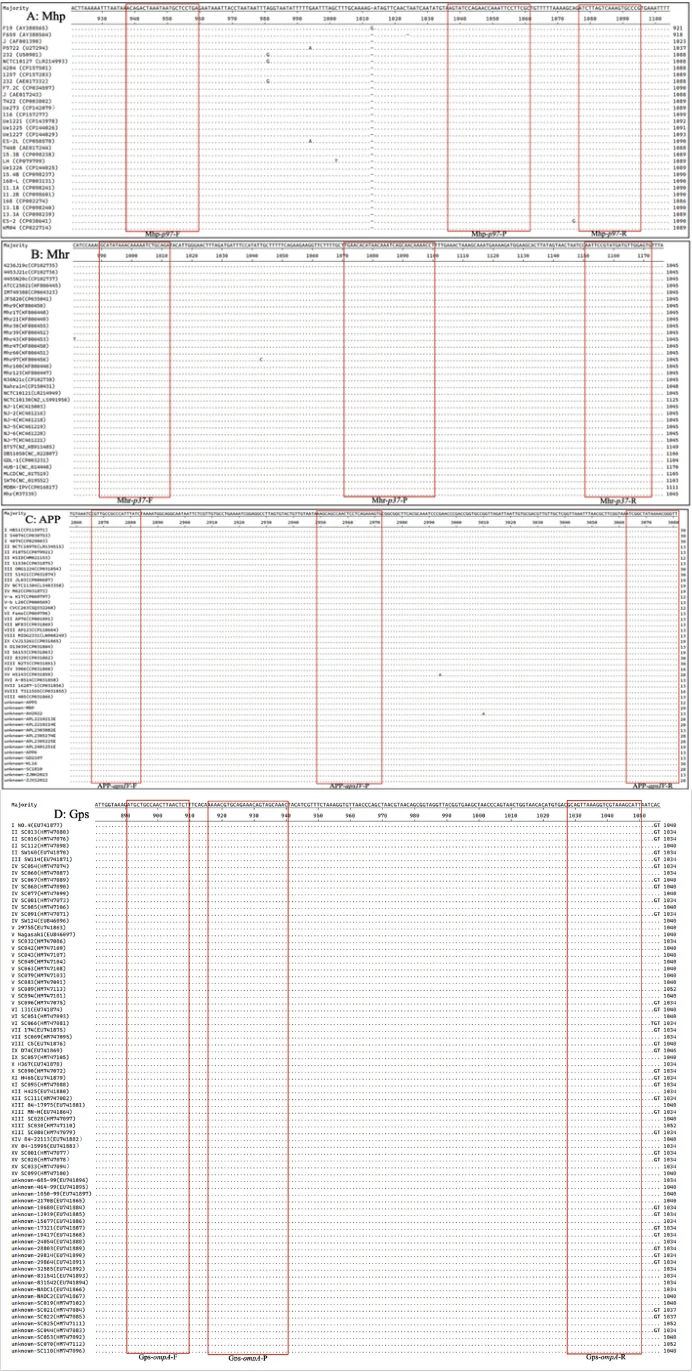
Primers and probes used in the quadruplex quantitative polymerase chain reaction assay. (A) Locations of the primers and probe within the nucleotide sequence alignment of the *p97* gene of Mhp. (B) Locations of the primers and probe within the nucleotide sequence alignment of the *p37* gene of Mhr. (C) Locations of the primers and probe within the nucleotide sequence alignment of the *apxIV* gene of APP. (D) Locations of the primers and probe within the nucleotide sequence alignment of the *ompA* gene of Gps. F = Forward primer; P = TaqMan probe; R = Reverse primer; Mhp = *Mycoplasma hyopneumoniae*; Mhr = *Mycoplasma hyorhinis*; APP = *Actinobacillus pleuropneumoniae*; Gps = *Glaesserella* *parasuis*.

Nasal swab samples were processed according to the China National Technical Standard, Specification of nucleic acid extraction used for high-throughput detection of pathogenic microorganisms (GB/T 40458-2021) (https://openstd.samr.gov.cn/bzgk/std/newGbInfo?hcno=D04700AFE3F8AD1A0DAF727D212F9730; accessed February 13, 2025). Briefly, each swab was rotated and pressed against the tube wall 5–6 times before being discarded. The remaining sample was transferred to a 2.0 mL centrifuge tube and centrifuged at 13,500 × *g* for 2 min at 4°C. The supernatant was removed, leaving approximately 50 μL of residual liquid. Subsequently, 400 μL of PBS (pH 7.2) was added, and the sample was thoroughly resuspended by pipetting before nucleic acid extraction.

A 200 μL aliquot of each processed clinical sample and vaccine solution was subjected to total nucleic acid extraction using the GeneRotex 96 Automatic Nucleic Acid Extractor (Tianlong, Xi'an, China) together with the Viral DNA/RNA Extraction Kit Ver. 4.0 (Catalog No. T324; Tianlong, Xi'an, China) according to the manufacturer's instructions. Nucleic acids were eluted in 80 μL of elution buffer.

The purity of the extracted nucleic acids was assessed using a NanoDrop spectrophotometer (Thermo Fisher Scientific, Waltham, MA, USA). Samples with A260/A280 ratios of 1.8-2.0 and A260/A230 ratios of 1.9-2.2 were considered suitable for subsequent qPCR analysis. Extracted nucleic acids were either analyzed immediately or stored at −80°C for no longer than 48 h before analysis.

### Construction of the standard plasmids

Nucleic acids from Mhp, Mhr, APP, and Gps were extracted from vaccine preparations or positive clinical samples. The target fragments corresponding to the *p97* gene of Mhp, the *p37* gene of Mhr, the *apxIV* gene of APP, and the *ompA* gene of Gps were amplified by PCR using the primers listed in Table 1.

The amplified products were purified using the MiniBEST DNA Fragment Purification Kit Ver. 4.0 (Code No. 9761; TaKaRa, Dalian, China), ligated into the pMD18-T vector (Code No. 6011; TaKaRa, Dalian, China), and transformed into DH5α competent cells (Code No. 9057; TaKaRa, Dalian, China). Positive clones were selected and cultured at 37°C for approximately 20 h. Recombinant plasmids were subsequently extracted using the MiniBEST Plasmid Extraction Kit Ver. 4.0 (Code No. 9760; TaKaRa, Dalian, China). The resulting recombinant plasmids were designated p-Mhp, p-Mhr, p-APP, and p-Gps and were used as standard plasmids throughout the study.

Plasmid concentrations were determined using a NanoDrop spectrophotometer (Thermo Fisher Scientific, Waltham, MA, USA). The OD260 values were measured, and plasmid concentrations (ng/μL) were calculated. Plasmid copy numbers were subsequently determined using the following formula:


\[
Plasmid copy number (copies/\mu L)=\frac{Plasmid concentration (ng/\mu L)\times {10}^{-9}\times 6.02\times {10}^{23}}{660\times Plasmid length (bp)}
\]

### Optimization of reaction conditions

The quadruplex qPCR assay was optimized using an ABI QuantStudio™ 5 Real-Time PCR System (Thermo Fisher Scientific, Waltham, MA, USA). Amplification data were analyzed using QuantStudio™ Design & Analysis Software version 1.5.3 (Thermo Fisher Scientific).

The four recombinant standard plasmids were mixed in equal proportions and diluted to 1 × 10⁸ copies/μL for use as templates. Reaction conditions were optimized using a comprehensive checkerboard design to ensure robust performance in complex clinical matrixes. Each 20 μL reaction contained 10 μL of 2× Premix Ex Taq™ (Probe qPCR) (Code No. RR390A; TaKaRa, Dalian, China), 0.1-0.6 μL each of primers and probes (20 μM), 2.0 μL of the mixed standard plasmids (1 × 10⁸ copies/μL), and nuclease-free water (Code No. 9012; TaKaRa, Dalian, China) to a final volume of 20 μL.

Optimization was performed in two steps. First, primer concentrations (20 μM; 0.2-0.6 μL) and probe concentrations (20 μM; 0.1-0.4 μL) were independently optimized at a fixed annealing temperature of 58°C. The optimal primer and probe concentrations were selected based on the lowest cycle threshold (Ct) values and the highest fluorescence intensity (ΔRn). Subsequently, using the optimized primer and probe concentrations, annealing temperatures ranging from 55°C to 62°C were evaluated. The optimal annealing temperature was selected according to the lowest Ct value and the highest ΔRn value.

### Generation of the standard curves

The standard plasmids p-Mhp, p-Mhr, p-APP, and p-Gps were mixed in equal proportions and serially diluted 10-fold from 1 × 10⁸ to 1 × 10² copies/μL, corresponding to final reaction concentrations of 1 × 10⁷ to 1 × 10¹ copies/µL. These plasmid mixtures were used to generate the standard curves for the quadruplex qPCR assay.

### Analytical specificity

Analytical specificity was evaluated using nucleic acids extracted from CSFV, PCV2, PRRSV, PRV, SIV, Mhp, Mhr, APP, Gps, *Escherichia coli*, group C *Streptococcus*, *Streptococcus suis* serotype 2, *Salmonella choleraesuis*, *Erysipelothrix rhusiopathiae*, and *Pasteurella multocida*. The recombinant plasmids (p-Mhp, p-Mhr, p-APP, and p-Gps), positive clinical samples, and nuclease-free distilled water were included as positive and negative controls.

### Analytical sensitivity

The standard plasmids p-Mhp, p-Mhr, p-APP, and p-Gps were mixed in equal proportions and serially diluted 10-fold from 1 × 10⁸ to 1 × 10⁰ copies/μL, corresponding to final reaction concentrations of 1 × 10⁷ to 1 × 10⁻¹ copies/µL. The diluted plasmid mixtures were amplified under the optimized reaction conditions to evaluate the analytical sensitivity of the assay. Each concentration was tested in triplicate.

To determine the limit of detection (LOD), the four standard plasmids were mixed in equal proportions and diluted to final concentrations of 500, 250, 125, 100, 62.5, and 50 copies/reaction. Each concentration was tested in 24 replicates under the optimized reaction conditions, and Ct values were recorded. Probit regression analysis was performed using IBM SPSS Statistics version 26 (IBM Corp., Armonk, NY, USA; accessed February 13, 2025) to estimate the LOD. The use of 24 replicates per concentration was based on experimental design principles to ensure reliable estimation while maintaining experimental feasibility.

### Repeatability analysis

The standard plasmids p-Mhp, p-Mhr, p-APP, and p-Gps were mixed in equal proportions and serially diluted 10-fold. Final reaction concentrations of 1.0 × 10⁷, 1.0 × 10⁵, and 1.0 × 10³ copies/µL were used to evaluate assay repeatability. Three independent experiments were performed on different days, with three replicates at each concentration. Ct values were recorded, and intra- and inter-assay coefficients of variation (CVs) were calculated.

### Detection of clinical samples

A total of 1,238 clinical samples, comprising 821 lung tissue samples and 417 nasal swab samples collected from 14 cities in Guangxi Province, China, were analyzed using the quadruplex qPCR assay developed in this study to evaluate its clinical applicability.

For comparison, all samples were also tested using previously published qPCR assays for Mhp and Mhr [20], Gps [21], and the qPCR assay for APP recommended in the China Agricultural Industry Standard, Diagnostic techniques for porcine contagious pleuropneumonia (NY/T537-2023) (https://std.samr.gov.cn/hb/search/ stdHBDetailed?id=F997A516CBA31C94E05397BE0A0A70DA; accessed February 13, 2025). Diagnostic sensitivity, diagnostic specificity, and agreement rates of the developed quadruplex qPCR assay were calculated according to the method described by Mo *et al.* [22]. The primer and probe sequences and target genes used in the reference assays are provided in Supplementary Table S7.

### Statistical analysis

Detection data were analyzed using Microsoft Excel 2019 (https://www.microsoft.com; accessed February 13, 2025) and IBM SPSS Statistics version 26 (IBM Corp., Armonk, NY, USA; https://www.ibm.com/products/spss-statistics; accessed February 13, 2025).

Diagnostic sensitivity, diagnostic specificity, and agreement rates were calculated according to the formulas reported by Mo *et al.* [22]. Agreement rates were compared using the chi-square test, with p < 0.05 considered statistically significant. The 95% CIs for diagnostic sensitivity, diagnostic specificity, and agreement rates were calculated using the Wilson score method.

## RESULTS

### Construction of the standard plasmids

The target fragments corresponding to the *p97* gene of Mhp, the *p37* gene of Mhr, the *apxIV* gene of APP, and the *ompA* gene of Gps were amplified by PCR, purified, and used to construct recombinant standard plasmids. The resulting plasmids were designated p-Mhp, p-Mhr, p-APP, and p-Gps, with initial concentrations of 4.46 × 10¹⁰, 3.48 × 10¹⁰, 2.85 × 10¹⁰, and 3.20 × 10¹⁰ copies/μL, respectively. The recombinant plasmids were verified by Sanger sequencing (IGE Biotechnology, Guangzhou, China) using M13 primers, confirming the correctness of the inserted target gene sequences (Supplementary Table S8). All recombinant plasmids were diluted to 1 × 10¹⁰ copies/μL and stored at −80°C until use.

### Determination of the optimal reaction conditions

The optimal reaction conditions were established using a checkerboard optimization approach by evaluating annealing temperatures, primer and probe concentrations, and the number of amplification cycles. The optimized 20 μL reaction mixture is presented in Table 2.

The optimized amplification program consisted of an initial denaturation at 95°C for 30 s, followed by 40 cycles of denaturation at 95°C for 5 s and annealing/extension at 60°C for 30 s. Fluorescence signals were automatically recorded at the end of each amplification cycle. Samples with Ct values ≤35 were considered positive.

**Table 2:** Optimal reaction mixture for the quadruplex quantitative polymerase chain reaction assay.

**Reagent**	**Volume (** **μL** **)**	**Final concentration (** **nM** **)**
Premix Ex Taq™ (2×)	10.0	1×
Mhp-p97-F (20 pmol/μL)	0.4	400
Mhp-p97-R (20 pmol/μL)	0.4	400
Mhp-p97-P (20 pmol/μL)	0.3	300
Mhr-p37-F (20 pmol/μL)	0.2	200
Mhr-p37-R (20 pmol/μL)	0.2	200
Mhr-p37-P (20 pmol/μL)	0.2	200
APP-apxIV-F (20 pmol/μL)	0.2	200
APP-apxIV-R (20 pmol/μL)	0.2	200
APP-apxIV-P (20 pmol/μL)	0.2	200
Gps-ompA-F (20 pmol/μL)	0.3	300
Gps-ompA-R (20 pmol/μL)	0.3	300
Gps-ompA-P (20 pmol/μL)	0.3	300
Total nucleic acids	2.0	—
RNase-free distilled water	Up to 20	—

### Generation of the standard curves

Equal proportions of p-Mhp, p-Mhr, p-APP, and p-Gps were mixed and subjected to 10-fold serial dilution. Plasmid mixtures with final reaction concentrations ranging from 1.0 × 10⁷ to 1.0 × 10¹ copies/µL were used as templates to generate standard curves under the optimized qPCR conditions (Figure 2).

The standard curves demonstrated excellent amplification performance. The slopes, intercepts, amplification efficiencies (Eff%), and correlation coefficients (R²) were −3.342, 36.289, 99.16%, and 0.998 for Mhp; −3.332, 36.499, 99.56%, and 0.999 for Mhr; −3.289, 35.648, 101.39%, and 0.999 for APP; and −3.384, 37.458, 97.48%, and 0.998 for Gps, respectively.

**Figure 2:**
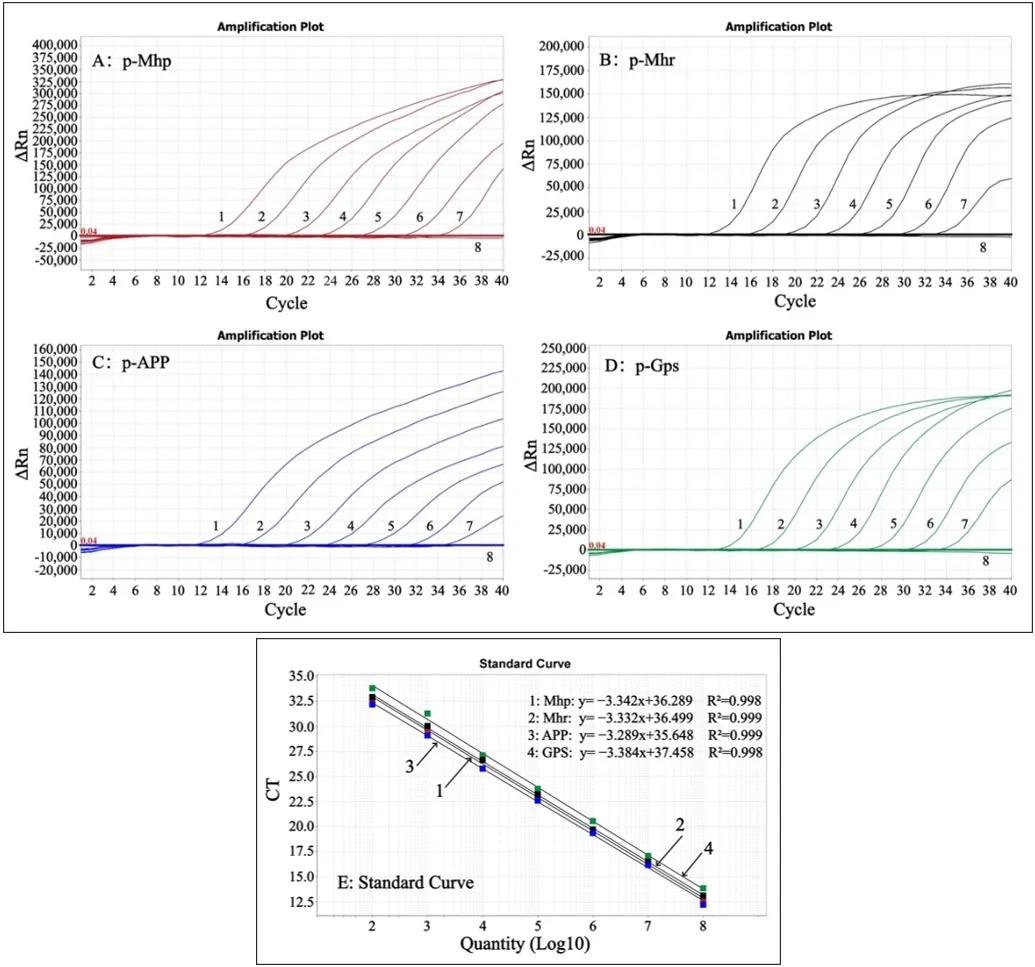
Amplification plots and corresponding standard curves of the quadruplex qPCR assay. (A) Amplification plots of p-Mhp. (B) Amplification plots of p-Mhr. (C) Amplification plots of p-APP. (D) Amplification plots of p-Gps. In (A–D), lanes 1–7 represent plasmid mixtures with final reaction concentrations ranging from 10⁷ to 10¹ copies/μL, and lane 8 represents the negative control. (E) Standard curves of the quadruplex qPCR assay; the x-axis represents the log₁₀ template copy number (copies/µL), and the y-axis represents the Ct value. Curves 1–4 correspond to Mhp, Mhr, APP, and Gps, respectively. qPCR = Quantitative polymerase chain reaction; Mhp = *Mycoplasma hyopneumoniae*; Mhr = *Mycoplasma hyorhinis*; APP = *Actinobacillus pleuropneumoniae*; Gps = *Glaesserella* *parasuis*; Ct = Cycle threshold.

### Analytical specificity

The analytical specificity of the quadruplex qPCR assay was evaluated using nucleic acids extracted from CSFV, PCV2, PRRSV, PRV, SIV, Mhp, Mhr, APP, Gps, *Escherichia coli*, group C *Streptococcus*, *Streptococcus suis* serotype 2, *Salmonella choleraesuis*, *Erysipelothrix rhusiopathiae*, and *Pasteurella multocida*. Specific amplification curves were observed only for Mhp, Mhr, APP, and Gps, whereas no amplification signals were detected from the remaining viral and bacterial pathogens (Figure 3), demonstrating the high analytical specificity of the developed assay.

**Figure 3:**
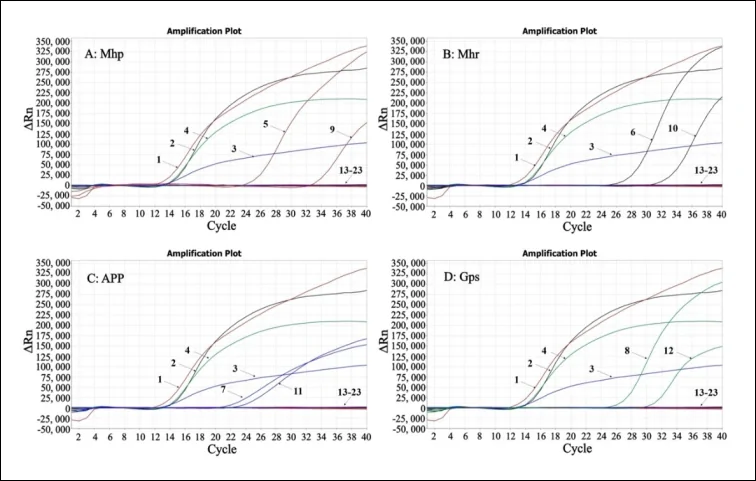
Analytical specificity of the quadruplex qPCR assay. (A) Analytical specificity for Mhp detected through the FAM channel (red). (B) Analytical specificity for Mhr detected through the ROX channel (black). (C) Analytical specificity for APP detected through the VIC channel (blue). (D) Analytical specificity for Gps detected through the Cy5 channel (green). In (A–D): 1 = p-Mhp recombinant plasmid; 2 = p-Mhr recombinant plasmid; 3 = p-APP recombinant plasmid; 4 = p-Gps recombinant plasmid; 5 = Mhp (strain J); 6 = Mhr (positive clinical sample); 7 = APP (trivalent vaccine containing serotype 1 strain JL9901, serotype 2 strain XT9904, and serotype 7 strain GZ9903); 8 = Gps (quadrivalent vaccine containing serotype 4 strain SD02, serotype 5 strain HN02, serotype 12 strain GZ01, and serotype 13 strain JX03); 9–12 = Positive clinical samples of Mhp, Mhr, APP, and Gps, respectively; 13 = CSFV; 14 = PCV2; 15 = PRRSV; 16 = PRV; 17 = SIV; 18 = *E. coli*; 19 = Group C *Streptococcus* and *S. suis* serotype 2; 20 = *S. choleraesuis*; 21 = *E. rhusiopathiae*; 22 = *P. multocida*; 23 = Nuclease-free distilled water. qPCR = Quantitative polymerase chain reaction; Mhp = *Mycoplasma hyopneumoniae*; Mhr = *Mycoplasma hyorhinis*; APP = *Actinobacillus pleuropneumoniae*; Gps = *Glaesserella* *parasuis*; FAM = 6-Carboxyfluorescein; ROX = Carboxy-X-rhodamine; VIC = VIC fluorescent dye; Cy5 = Cyanine 5; CSFV = Classical swine fever virus; PCV2 = Porcine circovirus type 2; PRRSV = Porcine reproductive and respiratory syndrome virus; PRV = Pseudorabies virus; SIV = Swine influenza virus.

Serial 10-fold dilutions of mixed recombinant plasmids (p-Mhp, p-Mhr, p-APP, and p-Gps) ranging from 1 × 10⁷ to 1 × 10⁻¹ copies/µL were used to evaluate analytical sensitivity. The assay successfully detected all four target pathogens at 10 copies/µL (Figure 4).

**Figure 4:**
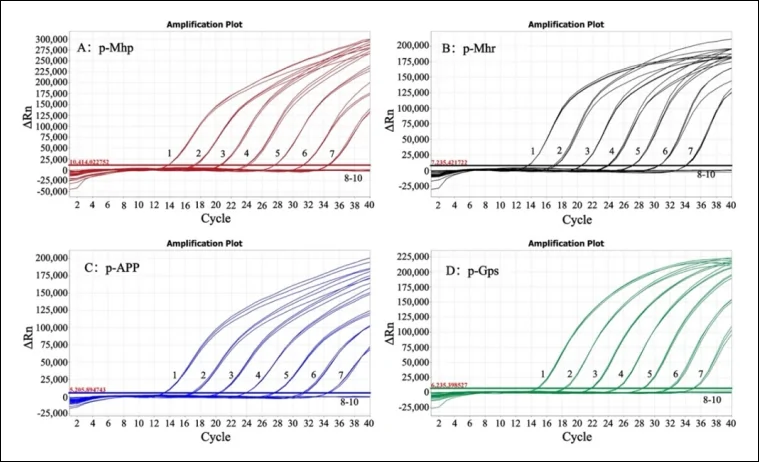
Analytical sensitivity of the quadruplex qPCR assay. (A) Analytical sensitivity for Mhp. (B) Analytical sensitivity for Mhr. (C) Analytical sensitivity for APP. (D) Analytical sensitivity for Gps. In (A–D), lanes 1–9 represent plasmid mixtures with final reaction concentrations ranging from 10⁷ to 10⁻¹ copies/µL, and lane 10 represents the negative control. qPCR = Quantitative polymerase chain reaction; Mhp = *Mycoplasma hyopneumoniae*; Mhr = *Mycoplasma hyorhinis*; APP = *Actinobacillus pleuropneumoniae*; Gps = *Glaesserella* *parasuis*.

To further determine the analytical LOD, recombinant plasmid mixtures were diluted to 500, 250, 125, 100, 62.5, and 50 copies/reaction and analyzed by Probit regression. The estimated LODs were 152.02 copies/reaction for p-Mhp, 158.53 copies/reaction for p-Mhr, 161.36 copies/reaction for p-APP, and 140.40 copies/reaction for p-Gps (Table 3**, **Figure 5).

**Table 3:** Ct values and detection rates of serially diluted recombinant standard plasmids.

**Plasmid**	**Final concentration (copies/reaction)**	**Number of samples**	**Ct value**	**Hit rate (%)**
p-Mhp	500	24	33.57	100
	250	24	34.62	100
	125	24	35.67	75.00
	100	24	35.77	37.50
	62.5	24	36.75	8.30
	50	24	ND	0
p-Mhr	500	24	33.95	100
	250	24	34.61	100
	125	24	35.65	66.67
	100	24	36.28	12.50
	62.5	24	ND	0
	50	24	ND	0
p-APP	500	24	33.51	100
	250	24	34.58	100
	125	24	35.10	70.84
	100	24	35.52	41.67
	62.5	24	36.95	16.67
	50	24	ND	0
p-Gps	500	24	33.98	100
	250	24	34.42	100
	125	24	35.46	83.33
	100	24	35.69	50.00
	62.5	24	35.70	8.30
	50	24	ND	0

qPCR = Quantitative polymerase chain reaction; Ct = Cycle threshold; ND = Not detected; Mhp = *Mycoplasma hyopneumoniae*; Mhr = *Mycoplasma hyorhinis*; APP = *Actinobacillus pleuropneumoniae*; Gps = *Glaesserella* *parasuis*.

**Figure 5:**
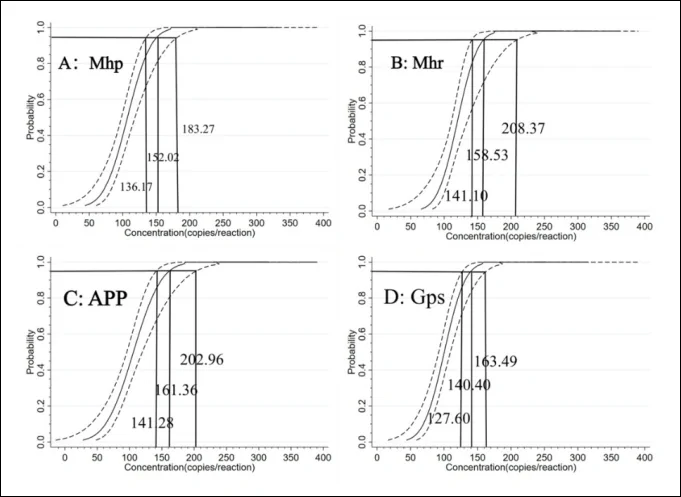
Determination of the analytical LOD of the quadruplex qPCR assay by Probit regression analysis. (A) Estimated LOD for Mhp = 152.02 copies/reaction. (B) Estimated LOD for Mhr = 158.53 copies/reaction. (C) Estimated LOD for APP = 161.36 copies/reaction. (D) Estimated LOD for Gps = 140.40 copies/reaction. LOD = Limit of detection; qPCR = Quantitative polymerase chain reaction; Mhp = *Mycoplasma hyopneumoniae*; Mhr = *Mycoplasma hyorhinis*; APP = *Actinobacillus pleuropneumoniae*; Gps = *Glaesserella* *parasuis*.

### Repeatability

The repeatability of the quadruplex qPCR assay was evaluated using mixed recombinant plasmids at final reaction concentrations of 1.0 × 10⁷, 1.0 × 10⁵, and 1.0 × 10³ copies/µL. The intra-assay CVs ranged from 0.44% to 2.17%, whereas the inter-assay CVs ranged from 0.49% to 2.44% (Table 4). All CVs were <2.50%, indicating excellent repeatability and reproducibility of the developed assay.

**Table 4:** Repeatability and reproducibility of the quadruplex quantitative polymerase chain reaction assay.

**Plasmid**	**Final concentration (copies/**µ**L)**	**Intra-assay mean Ct**	**Intra-assay SD**	**Intra-assay CV (%)**	**Inter-assay ** **mean** **Ct**	**Inter-assay SD**	**Inter-assay CV (%)**
p-Mhp	1.0 × 10⁷	12.39	0.15	1.21	12.42	0.16	1.29
	1.0 × 10⁵	19.95	0.12	0.60	19.94	0.13	0.65
	1.0 × 10³	26.74	0.14	0.52	26.73	0.16	0.60
p-Mhr	1.0 × 10⁷	11.05	0.24	2.17	11.06	0.27	2.44
	1.0 × 10⁵	18.50	0.17	0.92	18.50	0.19	1.03
	1.0 × 10³	25.60	0.15	0.59	25.61	0.17	0.66
p-APP	1.0 × 10⁷	12.46	0.12	0.96	12.46	0.11	0.88
	1.0 × 10⁵	20.36	0.11	0.54	20.37	0.11	0.54
	1.0 × 10³	27.53	0.13	0.47	27.61	0.14	0.51
p-Gps	1.0 × 10⁷	12.89	0.08	0.62	12.88	0.08	0.62
	1.0 × 10⁵	20.44	0.09	0.44	20.44	0.10	0.49
	1.0 × 10³	27.60	0.15	0.54	27.60	0.18	0.65

APP = *Actinobacillus pleuropneumoniae*; CV = coefficient of variation; Ct = cycle threshold; Gps = *Glaesserella* *parasuis*; Mhp = *Mycoplasma hyopneumoniae*; Mhr = *Mycoplasma hyorhinis*; qPCR = quantitative polymerase chain reaction; SD = standard deviation.

### Detection of clinical samples

A total of 1,238 clinical samples, comprising 821 lung tissue samples and 417 nasal swab samples collected in Guangxi Province, China, during 2025–2026, were tested using the developed quadruplex qPCR assay. The positivity rates were 31.91% (395/1,238) for Mhp, 18.09% (224/1,238) for Mhr, 4.20% (52/1,238) for APP, and 26.82% (332/1,238) for Gps (Table 5).

**Table 5:** Detection of pathogens and co-infections in clinical samples using the quadruplex quantitative polymerase chain reaction assay.

**Region**	**No. of samples**	**Mhp**	**Mhr**	**APP**	**Gps**	**P + R**	**P + A**	**P + G**	**R + A**	**R + G**	**A + G**	**P + R + A**	**P + R + G**	**P + A + G**	**R + A + G**	**P + R + A + G**
Nanning	89	36	7	0	19	1	0	1	0	5	0	0	0	0	0	0
Liuzhou	179	75	32	8	37	8	4	12	1	11	1	0	1	2	1	0
Guilin	28	17	7	0	6	1	0	0	0	5	0	0	1	0	0	0
Wuzhou	120	76	11	0	6	7	0	0	0	3	0	0	1	0	0	0
Beihai	50	3	8	0	20	0	0	1	0	7	0	0	0	0	0	0
Chongzuo	185	7	37	1	46	1	0	2	0	24	0	0	0	0	1	0
Laibin	50	11	24	0	27	0	0	1	0	23	0	0	1	0	0	0
Hezhou	141	62	27	8	34	3	0	2	0	13	0	0	4	1	4	1
Yulin	100	13	20	31	38	0	1	2	2	14	9	0	0	0	1	0
Baise	83	24	15	0	36	0	0	4	0	15	0	0	0	0	0	0
Hechi	42	12	3	0	3	1	0	0	0	1	0	0	0	0	0	0
Qinzhou	59	22	5	3	10	2	1	5	0	2	0	0	0	0	0	0
Fangchenggang	74	9	16	1	41	0	0	1	0	15	0	0	1	0	0	0
Guigang	38	28	12	0	9	4	0	0	0	8	0	0	0	0	0	0
Total	1,238	395	224	52	332	28	6	31	3	146	10	0	9	3	7	1
Positivity rate (%)	—	31.91	18.09	4.20	26.82	2.26	0.48	2.50	0.24	11.79	0.81	0	0.73	0.24	0.57	0.08

A = APP; APP = *Actinobacillus pleuropneumoniae*; G = Gps; Gps = *Glaesserella* *parasuis*; Mhp = *Mycoplasma hyopneumoniae*; Mhr = *Mycoplasma hyorhinis*; P = Mhp; qPCR = quantitative polymerase chain reaction; R = Mhr.

As shown in Table 6, the positivity rates among lung tissue samples were 46.77% (384/821) for Mhp, 9.99% (82/821) for Mhr, 1.34% (11/821) for APP, and 11.45% (94/821) for Gps. Among nasal swab samples, the corresponding positivity rates were 2.64% (11/417), 34.05% (142/417), 9.83% (41/417), and 57.07% (238/417), respectively. Chi-square analysis showed that the detection rates of all four pathogens differed significantly between lung tissue and nasal swab samples (Mhp: χ² = 250.82, p < 0.0001; Mhr: χ² = 112.97, p < 0.0001; APP: χ² = 49.68, p < 0.0001; Gps: χ² = 260.85, p < 0.0001).

Co-infections were also detected. Among lung tissue samples, the most frequently detected dual-infection was Mhp and Gps, with a positivity rate of 3.65% (30/821). Among nasal swab samples, the most frequently detected dual-infection was Mhr and Gps, with a positivity rate of 29.02% (121/417).

All 1,238 samples were also tested in parallel using the reference assays. The positivity rates obtained using the reference assays were 32.55% (403/1,238) for Mhp, 17.93% (222/1,238) for Mhr, 3.96% (49/1,238) for APP, and 27.38% (339/1,238) for Gps. Discordant results between the developed and reference assays were recorded, and the diagnostic sensitivity and specificity of the developed assay were calculated.

The diagnostic sensitivity and specificity were 96.77% and 99.40% for Mhp, 95.95% and 98.92% for Mhr, 95.92% and 99.58% for APP, and 95.87% and 99.22% for Gps, respectively (Table 7). The agreement rates between the developed and reference assays were 98.55% for Mhp, 98.38% for Mhr, 99.43% for APP, and 98.30% for Gps (Table 8).

**Table 6:** Detection of pathogens and co-infections according to sample source and sample type.

**Category**	**No. of samples**	**Mhp**	**Mhr**	**APP**	**Gps**	**P + R**	**P + A**	**P + G**	**R + A**	**R + G**	**A + G**	**P + R + A**	**P + R + G**	**P + A + G**	**R + A + G**	**P + R + A + G**
Slaughterhouse	966	387	126	11	157	27	6	30	1	72	1	0	6	2	1	0
Pig farm	272	8	98	41	175	1	0	1	2	74	9	0	3	1	6	1
Total	1,238	395	224	52	332	28	6	31	3	146	10	0	9	3	7	1
Positivity rate (%)	—	31.91	18.09	4.20	26.82	2.26	0.48	2.50	0.24	11.79	0.81	0	0.73	0.24	0.57	0.08
Lung tissue	821	384	82	11	94	27	6	30	1	25	1	0	5	2	1	0
Nasal swab	417	11	142	41	238	1	0	1	2	121	9	0	4	1	6	1
Total	1,238	395	224	52	332	28	6	31	3	146	10	0	9	3	7	1
Positivity rate (%)	—	31.91	18.09	4.20	26.82	2.26	0.48	2.50	0.24	11.79	0.81	0	0.73	0.24	0.57	0.08

A = APP; APP = *Actinobacillus pleuropneumoniae*; G = Gps; Gps = *Glaesserella* *parasuis*; Mhp = *Mycoplasma hyopneumoniae*; Mhr = *Mycoplasma hyorhinis*; P = Mhp; qPCR = quantitative polymerase chain reaction; R = Mhr.

**Table 7:** Diagnostic sensitivity and specificity of the quadruplex quantitative polymerase chain reaction assay.

**Target**	**Developed assay result**	**Reference assay positive**	**Reference assay negative**	**Total**	**Diagnostic sensitivity, % (95% CI)**	**Diagnostic specificity, % (95% CI)**
Mhp	Positive	390	5	395	96.77 (95.04–98.50)	99.40 (98.88–99.92)
	Negative	13	830	843		
	Total	403	835	1,238		
Mhr	Positive	213	11	224	95.95 (93.35–98.55)	98.92 (98.28–99.56)
	Negative	9	1,005	1,014		
	Total	222	1,016	1,238		
APP	Positive	47	5	52	95.92 (90.38–100.00)	99.58 (99.21–99.95)
	Negative	2	1,184	1,186		
	Total	49	1,189	1,238		
Gps	Positive	325	7	332	95.87 (93.75–97.99)	99.22 (98.64–99.80)
	Negative	14	892	906		
	Total	339	899	1,238		

APP = *Actinobacillus pleuropneumoniae*; CI = confidence interval; Gps = *Glaesserella* *parasuis*; Mhp = *Mycoplasma hyopneumoniae*; Mhr = *Mycoplasma hyorhinis*; qPCR = quantitative polymerase chain reaction.

**Table 8:** Agreement between the developed quadruplex quantitative polymerase chain reaction assay and the reference assays.

**Method**	**Mhp**	**Mhr**	**APP**	**Gps**
Developed assay	395/1,238	224/1,238	52/1,238	332/1,238
Reference assay	403/1,238	222/1,238	49/1,238	339/1,238
Agreement rate (%)	98.55	98.38	99.43	98.30

APP = *Actinobacillus pleuropneumoniae*; Gps = *Glaesserella* *parasuis*; Mhp = *Mycoplasma hyopneumoniae*; Mhr = *Mycoplasma hyorhinis*; qPCR = quantitative polymerase chain reaction.

## DISCUSSION

### Global epidemiological importance of the target pathogens

PRDC remains a major disease challenge affecting the global swine industry [2]. Mhp, Mhr, APP, and Gps are important bacterial pathogens that contribute directly or indirectly to PRDC development [13]. These pathogens have been detected at high rates in pig populations worldwide and pose a considerable threat to swine health and production [23, 24]. In Brazil, the seropositivity rates of Mhp and APP among slaughtered pigs were 88.40% and 5.70%, respectively [25]. In India, Mhr was detected in 21.33% of lung samples collected from slaughtered pigs [26]. In Poland, examination of 253 lung samples from wild boars revealed positivity rates of 41.90%, 3.95%, and 28.85% for Mhp, Mhr, and Gps, respectively. At least one of these pathogens was detected in 62.85% of the wild boars, whereas APP was not detected [27].

In China, the overall prevalence of Gps was 27.80% during 2005–2019 [9] and increased to 52.10% during 2022–2024 [28]. The pooled prevalence of Mhp during 2003–2024 was 33.40%, whereas clinically affected farms had a positivity rate of 52.90% [7]. Among clinically healthy pigs in eastern China during 2017–2019, the detection rates of APP and Gps were 0.45% and 33.30%, respectively [11]. In 2022, Mhr was detected in 31.77% of pig herds in China [8]. Collectively, these findings indicate that Mhp, Mhr, APP, and Gps are widely distributed among domestic pigs and wild boars worldwide. Therefore, an efficient and accurate assay that can simultaneously detect and differentiate these four pathogens is important for precise clinical diagnosis and effective disease control.

### Analytical performance of the quadruplex qPCR assay

Multiplex qPCR assays capable of detecting one to three of these pathogens have been reported [17, 20, 29]; however, no previous study has incorporated all four major porcine respiratory pathogens into a single detection system. To the best of our knowledge, this is the first quadruplex qPCR assay capable of simultaneously detecting and differentiating Mhp, Mhr, APP, and Gps in a single reaction.

In this study, four pairs of species-specific primers and probes were designed based on multiple sequence alignments of 31 Mhp, 35 Mhr, 46 APP, and 78 Gps target gene sequences obtained from the NCBI GenBank database. The primers and probes targeted conserved regions of the *p97*, *p37*, *apxIV*, and *ompA* genes, respectively, to provide broad coverage of available strains. Following systematic optimization of the reaction conditions, a rapid, specific, and sensitive quadruplex qPCR assay was established.

The assay specifically detected Mhp, Mhr, APP, and Gps without cross-reactivity with the other tested swine pathogens. Probit regression analysis showed LODs of 152.02, 158.53, 161.36, and 140.40 copies/reaction for Mhp, Mhr, APP, and Gps, respectively, corresponding to 7.60, 7.93, 8.07, and 7.02 copies/μL. These values were lower than the LOD of 10 copies/μL reported for the reference assays [20, 21], indicating greater analytical sensitivity under the experimental conditions used in this study. Intra-assay CVs ranged from 0.44% to 2.17%, and inter-assay CVs ranged from 0.49% to 2.44%, demonstrating good repeatability and reproducibility.

Compared with the reference methods, the developed assay showed diagnostic sensitivities of ≥95.87%, diagnostic specificities of ≥98.92%, and agreement rates of ≥98.30%. These findings indicate that the developed assay achieved diagnostic performance comparable to that of the reference singleplex assays while providing the additional advantage of simultaneous detection of four pathogens.

### Methodological advantages of simultaneous detection

This study presents the first quadruplex real-time qPCR assay for the simultaneous detection of four major bacterial pathogens associated with PRDC. Unlike previously reported duplex [20] or triplex [17, 21] assays, the developed method integrates Mhp, Mhr, APP, and Gps into a single reaction.

Compared with separate singleplex qPCR assays, the quadruplex assay can be completed within approximately 2 h and reduces reagent consumption by approximately 75%. It also reduces instrument occupancy time, labor requirements, and the risk of operational errors associated with repetitive testing. Furthermore, simultaneous detection reduces the likelihood that mixed infections involving Mhp, Mhr, APP, and Gps will be overlooked.

### Detection of pathogens in clinical samples

The clinical applicability of the developed assay was evaluated using 1,238 samples, comprising lung tissue and nasal swab samples collected from slaughterhouses and pig farms in 14 cities of Guangxi Province. The overall positivity rates were 31.91% (395/1,238) for Mhp, 18.09% (224/1,238) for Mhr, 4.20% (52/1,238) for APP, and 26.82% (332/1,238) for Gps.

Among lung tissue samples, the positivity rates were 46.77% for Mhp, 9.99% for Mhr, 1.34% for APP, and 11.45% for Gps. Among nasal swab samples, the corresponding rates were 2.64%, 34.05%, 9.83%, and 57.07%, respectively. The positivity rate of Mhp was significantly higher in lung tissue samples than in nasal swab samples (46.77% vs. 2.64%; p < 0.0001). This finding was consistent with that reported by Fablet *et al.* [30] and supports the suitability of lung tissue for detecting Mhp.

Although the positivity rate of Mhp in lung tissue samples in this study was lower than the 77.0% reported in Shanxi Province [31], it remained relatively high compared with other epidemiological data from China [32]. This finding suggests that Mhp remains an important respiratory pathogen in the sampled pig populations of Guangxi Province.

Mhr and Gps were detected significantly more frequently in nasal swab samples than in lung tissue samples (Mhr: 34.05% vs. 9.99%; Gps: 57.07% vs. 11.45%; p < 0.05). This result is consistent with their reported colonization of the upper respiratory tract [33, 34] and indicates that sample type should be selected according to the target pathogen.

The overall positivity rate of Gps in this study was 26.82%, which was comparable to the previously reported national prevalence of 27.80% in China [9]. However, the positivity rate of Gps in nasal swab samples was 57.07%, which was slightly higher than the 52.10% reported in eastern China but lower than the 71.76% reported in Jiangxi Province [28]. These differences may reflect variations in sampling strategy, study population, geographic region, and season [28].

APP was detected in 1.34% of lung tissue samples and 9.83% of nasal swab samples. These relatively low detection rates may be related to the preferential colonization of APP in the deep crypts of the tonsils and its detection during active disease or bacterial shedding [35]. The detection levels observed in this study were comparable to prevalence data reported in other countries [11, 25, 27], suggesting that APP occurred at a relatively low frequency in the sampled pig populations.

Rao *et al.* [16] reported detection rates of 48.19% for Gps and 42.47% for APP among pigs with respiratory signs in Guangxi Province. The differences between their findings and those of the present study may be related to differences in the sampled populations. Rao *et al.* [16] specifically examined pigs with respiratory signs and farms with a history of APP outbreaks, whereas the present study included samples collected on different dates from slaughterhouses and pig farms based on clinical signs and/or pathological lesions. These differences in sampling populations and inclusion criteria may have contributed to the variation in pathogen detection rates between the two studies.

### Mixed-infection patterns

Among the 1,238 clinical samples, 224 dual infections (18.09%), 19 triple infections (1.53%), and one quadruple infection (0.08%) were identified. Dual infections accounted for 91.80% (224/244) of all mixed infections. The most frequent dual-infection combination was Mhr + Gps (146 cases, 65.18%), followed by Mhp + Gps (31 cases, 13.84%) and Mhp + Mhr (28 cases, 12.50%).

The predominant dual-infection patterns differed between sample types. Mhr + Gps was the most frequent combination in nasal swab samples (121 cases), whereas Mhp + Gps was the most frequent combination in lung tissue samples (30 cases). The positivity rate of Mhr + Gps in nasal swab samples was significantly higher than that in lung tissue samples (29.02% [121/417] vs. 3.05% [25/821]; p < 0.0001).

Because Mhr and Gps can cause similar clinical manifestations, including polyserositis and arthritis, their frequent co-detection warrants further investigation of their potential interactions in PRDC-associated mixed infections. The developed assay enabled simultaneous identification of these mixed-infection patterns and demonstrated its practical value for detecting co-infections that may be overlooked when pathogens are tested separately.

### Study limitations

This study has several limitations. First, the clinical samples were collected primarily to evaluate the applicability of the developed quadruplex qPCR assay. Information on age, production category, vaccination status, clinical signs, and pathological lesions was recorded; however, data for some individual pigs, particularly the exact age and vaccination status of slaughtered pigs, were incomplete. Therefore, detailed analysis of associations between animal-level factors and pathogen positivity was limited.

Second, although the primers and probes were designed based on multiple sequence alignments and evaluated using BLAST analysis, genetic changes in emerging variants could result in mismatches and potential false-negative results. Continued monitoring of pathogen genetic variation and periodic evaluation of primer and probe sequences are therefore required.

Third, PCR inhibitors in clinical samples, including hemoglobin and polysaccharides, may affect amplification performance. Appropriate sample-processing and nucleic acid-purification procedures are needed to minimize matrix effects and reduce inhibition among different sample types.

Finally, the developed assay was validated in a single laboratory using clinical samples collected from Guangxi Province. Multicenter validation involving different laboratories and samples collected from other provinces of China is required to further evaluate its reproducibility, robustness, and broader applicability.

### Future applications

Multiplex nucleic acid detection technologies are increasingly being adapted for point-of-care applications [36]. Microfluidic and point-of-care testing platforms have also shown promise for detecting swine diseases [37, 38]. In future studies, the developed assay could be expanded to include additional respiratory pathogens and provide broader coverage of the PRDC-associated pathogen spectrum. Integration with microfluidic or point-of-care testing platforms may also facilitate its use on pig farms and during on-site epidemiological investigations, thereby supporting the prevention and control of animal diseases.

## CONCLUSION

A quadruplex qPCR assay was successfully developed and validated for the simultaneous detection of Mhp, Mhr, APP, and Gps, four major bacterial pathogens associated with PRDC. The assay demonstrated excellent analytical performance, with correlation coefficients (R²) ≥0.998; limits of detection of 152.02, 158.53, 161.36, and 140.40 copies/reaction for Mhp, Mhr, APP, and Gps, respectively; and high repeatability, with intra- and inter-assay CVs below 2.50%. Evaluation using 1,238 clinical samples showed positivity rates of 31.91%, 18.09%, 4.20%, and 26.82% for Mhp, Mhr, APP, and Gps, respectively. Compared with the reference assays, the developed method achieved diagnostic sensitivities of 95.87%–96.77%, diagnostic specificities of 98.92%–99.58%, and agreement rates exceeding 98%, confirming its excellent diagnostic performance.

The developed quadruplex qPCR assay provides a rapid, sensitive, and high-throughput approach for the simultaneous detection and differentiation of four clinically important bacterial pathogens in a single reaction. The ability to accurately identify mixed infections while substantially reducing reagent consumption, assay time, and laboratory workload makes this method well suited for routine veterinary diagnostic laboratories, epidemiological surveillance, outbreak investigations, and herd health monitoring. The observed differences in pathogen detection between lung tissue and nasal swab samples also provide practical guidance for selecting appropriate sample types according to the target pathogen.

A major strength of this study is the development of the first quadruplex qPCR assay capable of simultaneously detecting Mhp, Mhr, APP, and Gps in a single reaction. The assay integrates four important PRDC-associated bacterial pathogens into one diagnostic platform while maintaining high analytical sensitivity, specificity, repeatability, and agreement with established reference assays. Furthermore, validation using a large number of clinical samples collected from multiple cities demonstrated the robustness and practical applicability of the assay under field conditions.

Future studies should validate the assay using larger multicenter datasets from different geographical regions and pig production systems. Expanding the assay to include additional viral and bacterial pathogens involved in PRDC would further enhance its diagnostic value. Integration with automated, microfluidic, or point-of-care testing platforms could facilitate rapid on-farm diagnosis and real-time surveillance, thereby supporting more effective disease prevention and control strategies in the swine industry.

The developed quadruplex qPCR assay represents a reliable and efficient molecular diagnostic tool for the simultaneous detection of Mhp, Mhr, APP, and Gps. Its excellent analytical and diagnostic performance, combined with its ability to detect mixed infections in a single reaction, offers a practical solution for improving the diagnosis, surveillance, and management of PRDC. The assay has considerable potential for routine laboratory application and provides a valuable platform for future advances in multiplex molecular diagnostics for swine respiratory diseases.

## DATA AVAILABILITY

All data generated or analyzed during this study are included in this published article.

## GENERATIVE AI DECLARATION

The authors declare that no generative artificial intelligence (AI) tools or services were used in the research, writing, analysis, or any other aspect of this work.

## AUTHORS’ CONTRIBUTIONS

HW and YG: Contributed equally to this work. HW and YG: Investigation, methodology, formal analysis, and writing—original draft. KS and YW: Supervision, and writing—review and editing. YS, FL, and SF: Investigation and methodology. YY, WL, and SQ: Data curation, validation, and software. All authors have read and approved the final manuscript.
